# Complete mitochondrial genome of *Limnophyes minimus* (Diptera: Chironomidae)

**DOI:** 10.1080/23802359.2022.2029604

**Published:** 2022-01-27

**Authors:** Xiangliang Fang, Xintong Li, Tian Lu, Jun Fu, Mi Shen, Yunli Xiao, Yue Fu

**Affiliations:** Hubei Key Laboratory of Economic Forest Germplasm Improvement and Resources Comprehensive Utilization, Hubei Collaborative Innovation Center for the Characteristic Resources Exploitation of Dabie Mountains, Hubei Zhongke Research Institute of Industrial Technology, College of Biology and Agricultural Resources, Huanggang Normal University, Huanggang City, P.R. China

**Keywords:** Nematocera, *Limnophyes*, phylogenetic relationship

## Abstract

The complete mitochondrial genome of *Limnophyes minimus* (Meigen 1818) was sequenced and annotated, and its general features and base composition were analyzed. The phylogenetic relationships of the families Chironomidae, Simuliidae, Sciaridae and Culicidae based on 25 metagenomes were reconstructed using maximum likelihood (ML) methods based on the concatenated nucleotide sequences, the phylogenetic analysis showed that *L. minimus* belongs family Chironomidae, which is consistent with the traditional morphological classification.

*Limnophyes minimus* (Meigen 1818) belongs to the subfamily Orthocladiinae, is a widely distributed and recorded species in all six biogeographic regions. In China, the species is found in the Palearctic region (Ningxia, Tianjin, and Xinjiang) and Oriental region (Chongqing, Fujian, Guangxi, Guizhou, Hubei, Hunan, Jiangxi, Sichuan, Tibet, Taiwan, and Yunnan). Larva of this species can be found in various types of aquatic and semi-terrestrial environments (Sæther 1990; Wang 2000).

The specimen in this study was collected from Jingyang River (109°56′36″E, 30°20′39″N, alt. 677 m, Jianshi County, Enshi Tujia and Miao Autonomous Prefecture, Hubei Province, CHINA) on 6. VI. 2017, collected by Yue Fu (email: fuyue2007915@yahoo.com) and deposited in Biodiversity Herbarium of Huanggang Normal University (http://shengwu.hgnu.edu.cn/2018/1130/c435a7076/page.htm, Xiangliang Fang, wfs810806@163.com) under the voucher number HGNU-zzj02. The species was sequenced using Illumina Miseq platforms and annotated using the MITOS web server (Bernt et al. 2013). The mitochondrial genome was assembled using SPAdes version v3.11.1 (Bankevich et al. 2012). PhyloSuite (Zhang et al. 2020) was used for phylogenetic analyses with several plug-in programs: MAFFT (Katoh and Standley 2013) using ‘–auto’ strategy and codon alignment mode. PartitionFinder2 (Lanfear et al. 2017) was used to select best-fit partitioning schemes and models using the AICc criterion. Maximum likelihood phylogenies were inferred using IQ-TREE (Minh et al. 2013; Nguyen et al. 2015). The topology of the trees was visualized and edited in iTOL (Letunic and Bork 2019).

The complete mitogenome of *Limnophyes minimus* is 15,607 bp in size (GenBank accession number: MZ041033). It includes 13 protein-coding genes (PCGs), 22 tRNA genes, and two rRNA genes, a total of 37 genes, and one control region. There are 17 genes overlapping regions that appeared, with a total overlapping length of 52 bp, the longest overlapping region (9 bp) is located between trnW and trnC. There are 16 intergenic spacers with a total length of 427 bp, ranging from 1 bp to 118 bp. The longest intergenic is located between trnA and trnR. The genomic nucleotide composition is A: T: C: G = 40.09%: 37.59%: 13.02%: 9.30%. The total length of 13 PCGs in the mitochondrial genome is 11,179 bp. The initiation codons of PCGs comply with the ATN rule: there are four genes (nad2, atp8, nad5, nad6) with ATT as the start codon, seven genes (cox1, cox2, atp6, cox3, nad4, nad4l, and cob) with ATG as the start codon, one gene (nad3) with ATA as the start codon, and one gene (nad1) with TTG as the start codon. Except for cox1 with GTT as the stop codon, nad5 with TTT as the stop codon, nad4 with GAC as the stop codon, cob with CAC as the stop codon, others use TAA as the stop codon. The length of tRNA genes ranged from 65 bp to 73 bp, 1498 bp in total length. The length of 12S rRNA and 16S rRNA is 805 bp and 1373 bp in length, respectively.

Analysis of ML (Figure 1) showed that the family Chironomidae and Simuliidae were clustered together as sisters each other with at 98% bootstrap value of support, and both of these two families belong to Chironomoidea. Culicidae and Sciaridae + (Chironomidae + Simuliidae) were clustered together, which is generally consistent with previous phylogenetic analyses (Wood and Borkent 1989). The phylogenetic analysis showed that *Limnophyes minimus* is closely related to *Rheocricotopus villiculus* Wang and Sæther 2001, all belong to subfamily Orthocladiinae, which is in accordance with the traditional morphological classification.

**Figure 1.**
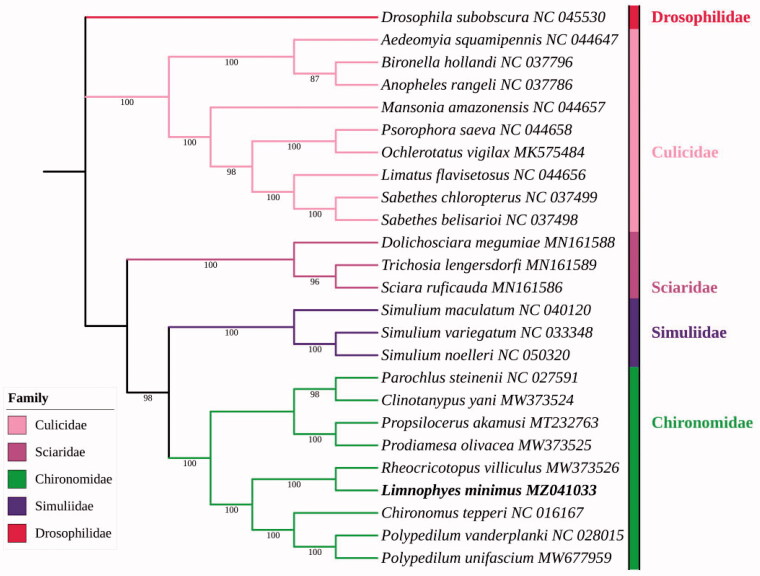
Phylogenetic tree based on 37 genes of mitogenomes of 25 Nematocera species inferred by maximum likelihood method (ML tree).

## Data Availability

The data that was newly obtained at this study are available in the NCBI under the accession number MZ041033 (https://www.ncbi.nlm.nih.gov/nuccore/MZ041033). The associated BioProject, SRA, and Bio-Sample numbers are PRJNA752840, SRR15368613, and SAMN20608202, respectively.
